# Socio-economic risk factors for intestinal helminthiases in selected endemic communities in Mindanao, the Philippines: a cross-sectional study

**DOI:** 10.1186/s12879-024-09780-5

**Published:** 2024-09-19

**Authors:** Vachel Gay V. Paller, Vicente Y. Belizario, Rico C. Ancog, Allen Jethro I. Alonte, Jasmine Renette D. Jimenez, Christina G. Corales, Billy P. Divina, Joaquin M. Prada, Martha Betson

**Affiliations:** 1 Institute of Biological Sciences, College of Arts and Sciences, University of the Philippines Los Baños, Laguna, The Philippines; 2 Department of Parasitology, College of Public Health, University of the Philippines Manila, Manila, The Philippines; 3 School of Environmental Science and Management, University of the Philippines Los Baños, Laguna, The Philippines; 4 Department of Veterinary Paraclinical Sciences, College of Veterinary Medicine, University of the Philippines Los Baños, Laguna, The Philippines; 5 Department of Comparative Biomedical Sciences, School of Veterinary Medicine, University of Surrey, Guildford, Surrey UK

**Keywords:** Neglected tropical diseases, Poverty, Health education, Schistosomiasis, Soil-transmitted helminthiasis

## Abstract

**Background:**

Parasitic neglected tropical diseases (NTDs) or ‘infectious diseases of poverty’ continue to affect the poorest communities in the world, including in the Philippines. Socio-economic conditions contribute to persisting endemicity of these infectious diseases. As such, examining these underlying factors may help identify gaps in implementation of control programs. This study aimed to determine the prevalence of schistosomiasis and soil-transmitted helminthiasis (STH) and investigate the role of socio-economic and risk factors in the persistence of these diseases in endemic communities in the Philippines.

**Methods:**

This cross-sectional study involving a total of 1,152 individuals from 386 randomly-selected households was conducted in eight municipalities in Mindanao, the Philippines. Participants were asked to submit fecal samples which were processed using the Kato-Katz technique to check for intestinal helminthiases. Moreover, each household head participated in a questionnaire survey investigating household conditions and knowledge, attitude, and practices related to intestinal helminthiases. Associations between questionnaire responses and intestinal helminth infection were assessed.

**Results:**

Results demonstrated an overall schistosomiasis prevalence of 5.7% and soil-transmitted helminthiasis prevalence of 18.8% in the study population. Further, the household questionnaire revealed high awareness of intestinal helminthiases, but lower understanding of routes of transmission. Potentially risky behaviors such as walking outside barefoot and bathing in rivers were common. There was a strong association between municipality and prevalence of helminth infection. Educational attainment and higher “practice” scores (relating to practices which are effective in controlling intestinal helminths) were inversely associated with soil-transmitted helminth infection.

**Conclusion:**

Results of the study showed remaining high endemicity of intestinal helminthiases in the area despite ongoing control programs. Poor socio-economic conditions and low awareness about how intestinal helminthiases are transmitted may be among the factors hindering success of intestinal helminth control programs in the provinces of Agusan del Sur and Surigao del Norte. Addressing these sustainability gaps could contribute to the success of alleviating the burden of intestinal helminthiases in endemic areas.

**Supplementary Information:**

The online version contains supplementary material available at 10.1186/s12879-024-09780-5.

## Background

Intestinal helminthiases, such as schistosomiasis and soil-transmitted helminthiasis (STH), are among the most common infectious parasitic diseases in the world. These parasitic neglected tropical diseases (NTDs) are also known as ‘infectious diseases of poverty’ for they continue to affect the poorest communities in the world, including in the Philippines. Intestinal helminthiases persist in areas with lack of access to safe water, sanitation, and hygiene (WASH) facilities, limited healthcare services, close proximity to animals, and military conflict zones [1–3]. These infections are not only caused by poverty, but they also contribute to poverty [2, 4, 5] .

Over one billion people worldwide are affected by these diseases [3]. Chronic or heavy intensity infections are associated with undernutrition, micronutrient deficiency, poor physical and cognitive development among children, and with decreased productivity in adults, leading to economic losses, exacerbating poverty [2, 6]. It is important to break this vicious cycle of parasites and poverty to effectively control and eliminate these intestinal helminthiases.

Global efforts are concerted to end the epidemic of NTDs by 2030 [7] in line with Sustainable Development Goal 3 [8]. Specific goals have been set for the different NTDs, with schistosomiasis and STH targeted for elimination as a public health problem [7]. Preventive chemotherapy is a core intervention strategy for schistosomiasis and STH. Improving access to WASH facilities and health education campaigns are also recommended for transmission control. The zoonotic nature of these intestinal helminths complicates control, thus implementation of complementary public health measures such as promotion of veterinary public health is suggested [1, 9]. In the Philippines, strategies including mass drug administration (MDA) and improving access to WASH facilities are being implemented [10–13]. However, despite these actions, intestinal helminthiases remain as significant public health problem in the country.

Macro- and micro- indicators such as programs and policies, socio-cultural practices, gender roles, and socio-economic status play a role in the transmission of intestinal helminths. Examining the underlying socio-economic aspects of diseases may identify limitations and gaps of control programs [14]. Findings of such studies could help in tailor fitting strategies to address identified challenges and strengthen the impact of control programs against these infections. In the Philippines, however, there are limited studies on the relationship between transmission of intestinal helminths and these factors, especially socio-economic and behavioral factors. Therefore, this present study determined the prevalence of schistosomiasis and STH and investigated the socio-economic and risk factors related to these infections in selected endemic communities in the Philippines, to investigate their contributions in the persistence of these parasitic infections.

## Methods

### Study sites and participants

The study was conducted in eight municipalities in the provinces of Agusan del Sur and Surigao del Norte in Caraga region, the Philippines in November 2019 and February 2021. Caraga is an administrative region in the northeastern part of Mindanao. A large proportion of the land in this region is used for raising crops, livestock, and other agricultural activities [15]. Among its five provinces, Agusan del Sur and Surigao del Norte are the major endemic foci of intestinal helminthiases, most especially schistosomiasis. Four municipalities from each province were selected based on the following criteria: known endemicity of intestinal helminths, willingness to cooperate with local government units (LGUs), accessibility of communities, and the security situation. Poverty characterized by overcrowding and limited access to WASH facilities is common in these endemic communities. Domesticated and farm animals which could serve as reservoirs of intestinal helminth infection are present. It is estimated that more than half of the households living in poverty are infected with intestinal helminths, although no mortality was recorded in the last 5 years.

This study was carried out as part of an interdisciplinary project, Zoonotic Transmission of Intestinal Parasites (ZooTRIP), which aimed to assess the contribution of zoonotic transmission and environmental reservoirs to the burden of human intestinal helminthiases in the Philippines. The sample size calculated based on the project objective was 1500 participants. As the average household size in the Philippines is five members [16], the targeted number of households was at least 300, with 37–38 in each municipality. The number of households needed per municipality was estimated using the ‘pwr’ package in R (version 3.4.4) [17], by evaluating the power to detect small, medium, and large effect sizes respectively (as defined by Cohen’s d). The power to detect medium and large effect sizes at a 95% significance level is over 90%, for small effects the power is 50%. Moreover, sampling 1500 participants allows the presence or absence of any disease to be determined if it can be detected with at least 40% sensitivity (and 100% specificity) with 95% confidence.

Households were randomly selected in each municipality and invited to participate in the study through the help of the LGUs. Members of the selected household ages 10 to 60 years old were included in the study and were asked to submit faecal samples for parasitological analysis.

### Study design

The study had a cross-sectional design. Parasitological assessment and a household survey were employed. The survey tools used in this study were designed based on consultation with researchers from University of the Philippines Los Baños, University of the Philippines Manila, and University of Surrey. The tools were written in English, translated into local languages, *Bisaya* and *Tagalog*. The tools were pre-tested in a different municipality in Agusan del Sur and Surigao del Norte after translation and modified accordingly based on the findings of pre-testing.

### Parasitological assessment

Stool containers were distributed by local health workers to study participants in the selected households. Stool samples were collected, transported to the field laboratory in a cold chain, processed using the Kato-Katz technique, and examined by trained field microscopists to determine the presence of *S. japonicum* and soil-transmitted helminths [18]. Two aliquots were prepared from each sample. The presence of other helminth ova was also noted.

The accuracy and reliability of the parasitological assessment were ensured through quality assurance measures such as use of freshly prepared reagents, appropriate laboratory techniques, meticulous examination of processed specimens, and accurate reporting of findings. All positive and 10% of all negative slides [18] were re-examined by a reference microscopist who was blinded to the initial results.

### Household survey

The developed questionnaire included questions related to (1) overall health status and past intestinal helminthiases, (2) knowledge and practices related to schistosomiasis and STH transmission and symptoms, and (3) household conditions, such as access to WASH facilities and exposure to animals. The questionnaire also covered relevant socio-economic characteristics of each household. Enumerators, who are fluent in *Bisaya*, *Tagalog*, and basic English, were trained by the research team to administer the questionnaire to the head of each household. In this study, household heads are the decision-makers in the family. In cases where both parents decide in the family, whoever was available was interviewed.

### Data processing and analysis

Data gathered were entered using Microsoft Excel 2010. As part of quality control, all data were re-checked for any errors in encoding and resolved as necessary by consulting the original document. For the household survey, ‘knowledge’ scores were calculated for each respondent with each correct answer related to transmission and symptoms of intestinal helminths scored with one point. Given some differences in transmission of intestinal helminths, ‘knowledge’ scores were calculated separately for schistosomiasis and STH. Total ‘knowledge’ scores were calculated as a sum of correct responses. Similarly, ‘practice’ scores were calculated, with one point awarded for every practice that is recognized to be effective in the prevention and control of intestinal helminths. Since good practices lead to prevention of any intestinal helminth disease, questions regarding practices were asked in relation to all intestinal helminthiases rather than individual diseases.

Data analysis was carried out using STATA version 16 (STATA 16^®^, Stata Corp, Texas, USA). Descriptive statistics was used for the sociodemographic characteristics of the households. The data was found to be non-normally distributed, as such, Spearman’s correlation was used to determine the relationship between ‘knowledge’ and ‘practice’ scores, and a Mann-Whitney U test was used to determine significant difference of knowledge scores between those households with infected individuals and households without infected individuals.

Logistic regression was also used to identify risk factors associated with intestinal helminthiases. Bivariate analysis of outcome and variables was carried out prior to the regression analysis. These variables include socio-economic characteristics, household conditions, ‘knowledge’ and ‘practice’ scores. Significant variables were used in the regression model. Backward stepwise multiple logistic regression analysis was done to identify predictive variables in the final model. In all analyses, a p-value of less than 0.05 was considered significant.

## Results

### Demography

A total of 386 households were enrolled in the study. Of these, 183 households were from Agusan del Sur and 203 households were from Surigao del Norte. Members of these enrolled households were invited to submit faecal samples for the parasitological assessment. A total of 1,152 individuals participated in the study. Among these, 566 individuals were from Agusan del Sur while 586 individuals were from Surigao del Norte and 537 were school-aged children (SAC) (i.e., ages 10–17 years old), while 615 were adults (i.e., ages 18 to 60 years old).

### Prevalence of intestinal helminthiases

Results of the parasitological assessment showed that 5.7% of the individuals (66/1,152, 95% Confidence Interval [95%CI]: 4.5–7.3) are infected with schistosomiasis. The prevalence in Surigao del Norte is significantly higher at 7.5% (44/586, 95%CI: 5.6–10.0) compared with Agusan del Norte at 3.8% (22/566, 95%CI: 2.5–5.9), (*p* = 0.012). Moreover, there was a significant variability in the prevalence among the municipalities with highest prevalence in San Isidro (20.1%) and lowest prevalence in Claver (2.9%), both from the province of Surigao del Norte (*p* < 0.001). When grouped by age, adults were found to have a prevalence of 4.9% (32/615, 95%CI:3.6–7.3) while SAC had a prevalence of 6.7% (34/537, 95%CI:4.5–8.8). No significant difference was seen between adults and SAC (*p* = 0.607).

Furthermore, the overall STH prevalence was 18.8% (217/1,152, 95%CI: 16.6–21.2) with significantly higher prevalence in Surigao del Norte at 24.9% (146/586, 95%CI: 21.6–28.7) compared to Agusan del Sur at 12.5% (71/566, 95%CI: 10.0-15.6), (*p* < 0.001). Similarly, STH prevalence was found to have significant variability across the municipalities (*p* < 0.001) with highest prevalence found again in San Isidro, Surigao del Norte (40.2%) and lowest prevalence in Claver, Surigao del Norte (7.9%). SAC was found to have significantly higher prevalence compared to adults at 22.2% (112/537, 95%CI: 17.5–24.6) and 16.2% (105/615, 95% CI: 14.2–20.3), respectively (*p* = 0.021).

The proportion of households with at least one member infected with schistosomiasis was 14.0% (54/386) while for STH 37.8% of the households (146/386) had at least one member infected.

### Socio-demographic characteristics of households

The household surveys revealed the sociodemographic and environmental characteristics of the selected households. The average household population size is approximately 6 members (range = 1–14). Farming is the major occupation of fathers (39.1%), while most mothers are unemployed (59.1%), with household monthly income averaging Php 7,016.00 (134.34 USD). Other types of employment include vendors, construction workers, or drivers. Table 1 summarises the socio-demographic characteristics of households.

**Table 1:** Socio-demographic characteristics of household heads and households surveyed in Agusan Del Sur (*N* = 183) and Surigao Del Norte (*N* = 203), the Philippines

Characteristics	Agusan del Sur		Surigao del Norte		Total	
	*n*	(%)	*n*	(%)	*n*	(%)
Gender						
Male	43	(23.5)	52	(25.6)	95	(24.6)
Female	140	(76.5)	151	(74.4)	291	(75.4)
Age						
Did not specify	19	(10.4)	50	(24.6)	69	(17.9)
19 to 34 years old	12	(6.6)	19	(9.4)	31	(8.0)
35 to 41 years old	56	(30.6)	41	(20.2)	97	(25.1)
42 to 48 years old	47	(25.7)	51	(25.1)	98	(25.4)
48 to 70 years old	49	(26.8)	42	(20.7)	91	(23.6)
Mean age (range)	45.0	(24–67)	44.0	(19–70)	44.5	(19–70)
Civil status						
Single	0	(0.0)	4	(2.0)	4	(1.0)
Married	145	(79.2)	161	(79.3)	306	(79.3)
Live-in	17	(9.3)	27	(13.3)	44	(11.4)
Widow/er	13	(7.1)	9	(4.4)	22	(5.7)
Others	8	(4.4)	2	(1.0)	10	(2.6)
Educational attainment						
No schooling	1	(0.6)	0	(0.0)	1	(0.3)
Primary level	68	(38.0)	72	(36.2)	140	(37.0)
Secondary level	84	(46.9)	102	(51.3)	186	(49.21)
Tertiary level	24	(13.4)	23	(11.6)	47	(12.4)
Vocational training	2	(1.1)	2	(1.0)	4	(1.1)
Household population						
1 to 5 members	98	(53.6)	95	(46.8)	193	(50.0)
6 to 10 members	80	(43.7)	99	(48.8)	179	(46.4)
More than 10 members	5	(2.7)	9	(4.4)	14	(3.6)
Mean population (range)	5.3	(1–14)	5.8	(1–12)	5.6	(1–14)
Occupation of father						
Unemployed	43	(23.5)	69	(34.0)	112	(29.0)
Farming	57	(31.2)	94	(46.3)	151	(39.1)
Others	83	(45.4)	40	(19.7)	123	(31.9)
Occupation of mother						
Unemployed	94	(51.4)	134	(66.0)	228	(59.1)
Farming	16	(8.7)	12	(5.9)	28	(7.3)
Laundry	14	(7.7)	11	(5.4)	25	(6.5)
Others	59	(32.2)	46	(22.7)	105	(27.2)
Mean household monthly income in Php (range)	6,452 (300 − 20,000)		7,510 (300 − 130,000)		7,016 (300 − 130,000)	
Overall family health status						
Generally sickly	95	(51.9)	103	(50.7)	198	(51.3)
Generally healthy	88	(48.1)	100	(49.3)	188	(48.7)
Previously diagnosed with any intestinal helminthiases	36	(19.7)	51	(25.1)	87	(22.5)

In terms of access to WASH facilities, purchasing water from refilling situation was reported as the major source of drinking water (48.0%) while use of community water systems is also common (21.8%). There were 18 households (4.7%) which had no own sanitary toilet at home, while 10 households (2.6%) had no access to toilets at all. The most common type of toilet was a pour-flush toilet (71.2%). The majority of households said that their water supply is adequate (89.6%). Furthermore, most households reported the presence of domestic animals (86.8%) and farm animals (52.3%) in the area. Only 89 households (23.1%) said that a slaughterhouse was near their house, while 337 households (87.3%) reported to have seen rodents around their area (see Table 2).

**Table 2:** Access to safe water, sanitation, and hygiene facilities and presence of animals of households surveyed in Agusan Del Sur (*N* = 183) and Surigao Del Norte (*N* = 203), the Philippines

Characteristics	Agusan del Sur		Surigao del Norte		Total	
	*n*	(%)	*n*	(%)	*n*	(%)
Source of drinking water						
Community water system	51	(28.3)	32	(15.9)	83	(21.8)
Deep well	14	(7.8)	3	(1.5)	17	(4.5)
Artesian well	17	(9.4)	27	(13.4)	44	(11.6)
Natural bodies of water	5	(2.8)	5	(2.5)	10	(2.6)
Rainwater	2	(1.1)	0	(0.0)	2	(0.5)
Water refilling station	73	(40.6)	110	(54.7)	183	(48.0)
Others	18	(10.0)	24	(11.9)	42	(11.0)
Source of water for domestic use						
Community water system	95	53.4	50	25.1	145	38.5
Deep well	37	20.8	18	9.1	55	14.6
Artesian well	15	8.4	62	31.2	77	20.4
Natural bodies of water	13	7.3	26	13.1	39	10.3
Rainwater	1	0.6	0	0.0	1	0.3
Others	17	9.6	43	21.6	60	15.9
Sanitary toilet at home						
Yes	176	(96.2)	192	(94.6)	368	(95.3)
No	7	(3.8)	11	(5.4)	18	(4.7)
Toilet ownership						
No toilet access	6	(3.3)	4	(2.0)	12	(3.1)
Owned	168	(91.8)	193	(95.1)	361	(93.5)
Shared	9	(4.9)	6	(2.9)	15	(3.9)
Type of sanitary toilet						
No toilet access	6	(3.3)	4	(2.0)	12	(2.6)
Flush toilet	6	(3.3)	4	(2.0)	10	(2.6)
Poor flush toilet	122	(66.7)	153	(75.3)	275	(71.2)
Pit latrine	48	(26.2)	42	(20.7)	90	(23.3)
Hanging latrine	1	(0.5)	0	(0.0)	1	(0.3)
Adequacy of water supply						
Adequate	133	(85.8)	176	(92.6)	309	(89.6)
Inadequate	22	(14.2)	14	(7.4)	36	(10.4)
Solid waste disposal						
Garbage collection	110	(60.1)	111	(54.7)	221	(57.3)
Composting	10	(5.5)	12	(5.9)	22	(5.7)
Burning	18	(9.8)	18	(8.9)	36	(9.3)
Pit	6	(3.3)	11	(5.4)	17	(4.4)
Throwing in lakes or rivers	0	(0.0)	1	(0.5)	1	(0.3)
Others	39	(21.3)	50	(24.6)	89	(23.1)
Presence of domestic animals						
Yes	153	(83.6)	182	(89.7)	335	(86.8)
No	30	(16.4)	21	(10.3)	51	(13.2)
Mean cat population (range)	0.6	(0–7)	0.7	(0–7)	0.7	(0–7)
Mean dog population (range)	1.1	(0–11)	1.0	(0–8)	1.1	(0–11)
Presence of farm animals						
Yes	74	(40.4)	110	(54.2)	202	(52.3)
No	109	(59.6)	93	(45.8)	184	(47.7)
Mean cattle population (range)	0.1	(0–2)	0.1	(0–4)	0.1	(0–4)
Mean water buffalo population (range)	0.1	(0–3)	0.3	(0–6)	0.2	(0–6)
Mean pig population (range)	0.9	(0–15)	0.9	(0–17)	0.9	(0–17)
Nearby slaughterhouse						
Yes	34	(18.6)	55	(27.1)	89	(23.1)
No	149	(81.4)	148	(72.9)	297	(76.9)
Presence of rodents						
Yes	160	(87.4)	177	(87.2)	337	(87.3)
No	23	(12.6)	26	(12.8)	49	(12.7)

### Knowledge, attitude, and practices on intestinal helminthiases

Table 3 summarizes the knowledge, attitudes, and practices in relation to intestinal helminthiases in the area. High awareness of the existence of intestinal helminthiases was seen in the area (88.1%). Most of the households were aware that medicines and diagnostic services are available in health centers. Many were also aware of health programs in their village with deworming programs as the most cited program.

**Table 3:** Knowledge, attitude, and practices in relation to intestinal helminthiases of households surveyed (*N* = 386) in Agusan Del Sur and Surigao Del Norte, the Philippines

Parameters	Schistosomiasis			STH		
	*n*	(%)		*n*		(%)
**Knowledge**						
Aware of intestinal helminthiases	340	(88.1)		340		(88.1)
Transmission						
Drinking contaminated water	61	(15.8)		64		(16.6)
Eating contaminated food	29	(7.5)		122		(31.6)
Eating raw or uncooked food	26	(6.7)		26		(6.7)
Frequent contact with soil	102	(26.4)		142		(36.8)
Frequent contact with water	162	(42.0)		38		(9.8)
Exposure to animals	20	(5.2)		15		(3.9)
No idea	18	(4.7)		17		(4.4)
Signs and symptoms						
Common cold, cough, fever	36	(9.3)		33		(8.6)
Difficulty in breathing	15	(3.9)		4		(1.0)
Headache, nausea	106	(27.5)		46		(11.9)
Weight loss, loss of appetite	40	(10.4)		47		(12.2)
Abdominal pain, vomiting	145	(37.6)		190		(49.2)
Diarrhea, bloody stool	67	(17.4)		39		(10.1)
Fatigue, body pain, weakness	33	(8.6)		19		(4.9)
Constipation, flatulence	52	(13.5)		37		(9.6)
Pruritus ani, rashes	8	(2.1)		11		(2.9)
Poor school performance	11	(2.9)		9		(2.3)
No idea	14	(3.6)		29		(7.5)
Mean ‘knowledge’ score (range)	2.8	(0–9)		3.3		(0–8)
Availability of medicine in health center	324	(83.9)		315		(81.6)
Availability of diagnostic test in health center	245	(63.5)		243		(63.0)
Awareness in village health programs ^a^	337				(87.31)	
**Attitude**						
Belief intestinal helminths are potentially fatal ^a^						
Yes	333				(86.3)	
No	53				(13.7)	
Belief intestinal helminths can be prevented and controlled						
Yes	290	(75.1)		302		(78.2)
No	96	(24.9)		84		(21.8)
Willingness to spend for treatment						
Yes	283	(73.3)		271		(70.2)
No	103	(26.7)		115		(29.8)
Willingness to vote for local ordinance to construct sanitary toilets ^a^						
Yes	359				(93.0)	
No	27				(7.0)	
**Practice**						
Walking outside barefoot ^a^						
Yes				223		(57.8)
No				163		(42.2)
Exposure to rice fields ^a^						
Yes				199		(51.6)
No				187		(48.4)
Bathe in rivers or irrigation channels ^a^						
Yes				175		(45.3)
No				211		(54.7)
Contact with snail-infested waters ^a^						
Yes				184		(47.7)
No				202		(52.3)
Practice handwashing before eating ^a^						
Yes				341		(88.3)
No				45		(11.7)
Habit of eating raw/undercooked food ^a^						
Yes				87		(22.5)
No				299		(77.5)
Participate in deworming ^a^						
Yes				355		(92.0)
No				31		(8.0)
Mean ‘practice’ score (range)				5.6		(2–9)
Treatment seeking behavior						
Do nothing	49	(12.7)		51		(13.2)
Self-medicate	4	(1.0)		13		(3.4)
Herbal medicine/traditional healers	3	(0.8)		14		(3.6)
Visit health centers/ hospital	320	(82.9)		297		(76.9)
Others	10	(2.6)		11		(2.9)
Severity of illness prior to seeking treatment						
Did not indicate	41	(10.6)		51		(13.2)
Mild	248	(64.3)		243		(63.0)
Moderate	77	(20.0)		75		(19.4)
Severe	20	(5.2)		17		(4.4)

^a^ Questions related to infections as a group

The most common understanding of transmission of schistosomiasis was frequent contact with water (42.0%), while frequent contact with soil was most common for STH (36.8%). Transmission through exposure to animals was not common knowledge (1.8–5.2%). The most common knowledge relating to signs and symptoms for these infections were abdominal pain and vomiting (37.8–49.2%). Generally low ‘knowledge’ scores were observed, with higher average scores seen in relation STH with 3.3 out of 9 (range = 0–8) than schistosomiasis with 2.8 out of 9 (range = 0–9).

Many respondents believed that intestinal helminthiases were potentially fatal when left untreated (86.3%), but that infections could be prevented and controlled (65.8–78.2%). The majority are also willing to pay for treatment (63.2–73.3%) and vote for local ordinance to construct sanitary toilets (93.0%). In terms of practices, most of the households said that they participate in deworming activities (92.0%) and practice handwashing before eating (88.3%). The results showed a mean ‘practice’ score of 5.6 out of 9 (range = 2–9).

There was a significant negative association between good practices and knowledge of schistosomiasis (rs (386) = -0.13, *p* = 0.008) and STH (rs (386) = -0.24, *p* < 0.001). The ‘knowledge’ scores of infected individuals were significantly higher than those uninfected (*p* < 0.001) for both schistosomiasis and STH. Furthermore, households willing to spend on treatment of intestinal helminths and to vote for local ordinance had higher ‘knowledge’ scores than those who do not (*p* < 0.001).

### Risk factors for intestinal helminthiases

Generalized mixed model analysis was done to determine risk factors related to intestinal helminthiases. The municipalities were considered as a random effect in all models. Results of the analysis showed that SAC were more likely to be infected with STH compared with adults (OR = 1.33; *p* = 0.032). Meanwhile for schistosomiasis, results of the analysis showed that females were less likely to be infected compared with males (OR = 0.55; *p* = 0.020).

Household level analysis showed that a higher ‘practice’ score was a protective factor against STH infection (OR = 0.83; *p* < 0.001). In contrast for schistosomiasis, exposure to rice fields were found to be a significant risk factor, where it increased the odds of being infected of up to almost four times (OR = 3.67; *p* < 0.001) (see Table 4). A complete table that includes non-statistically significant results is found in supplementary materials (Supplementary Table).

**Table 4:** Risk factors associated with intestinal helminth infection in Agusan Del Sur and Surigao Del Norte, the Philippines

Outcomes/Variables	Odds Ratio	Standard Error	*p* value
Risk factors associated to schistosomiasis*			
Sex (baseline males)			
*Females*	0.55	0.253	0.020
Exposure to rice fields	3.67	0.338	< 0.001
Risk factors associated to STH*			
Age Group (baseline adults)			
*School-age children*	1.33	0.136	0.008
‘Practice’ score *(continuous)*	0.83	0.055	< 0.001

^*^ *Municipality was considered as random effects in this model*

## Discussion

The interactions of people in poverty with parasites contribute to the persistence of both problems in the Philippines [2]. Programs to control these parasites and alleviate poverty have been implemented in the country for years [11, 12, 19]. However, they remain a problem in many communities. This study focused on intestinal helminthiases and examined socio-economic and risk factors to identify possible gaps in implementation of control programs in selected endemic communities in the Philippines.

Results of the parasitological assessment showed a higher prevalence of schistosomiasis compared to the global targets set by WHO [7]. There is no significant difference between age groups implying that residents in the area regardless of age are equally at risk. Similar to previous work [20], prevalence was found to be significantly higher in males compared with females. This may be because males are more involved in the agricultural sector resulting to frequent exposure to infested water. In contrast, findings of other study showed that females are at higher risk for schistosomiasis since they are exposed to infested water from carrying out domestic activities [21]. The social roles of males and females was found to be linked to infection, but the relationship vary depending on the social structure and roles of these genders in the community [1]. Furthermore, the results of regression analysis at the household level showed that exposure to rice field is a significant risk factor for schistosomiasis where it increases the likelihood of infection. This finding supports the previous statements wherein males are found to have higher prevalence compared with females. As expected, frequent contact with water would increase the risk for schistosomiasis, as such nature of employment can also influence the risk of exposure to intestinal helminthiasis where farmers and fisherfolks are more likely to be exposed to schistosomes compared with other occupations [20–24]. This poses a serious problem in the selected study sites since results of the survey revealed that farming is the most common occupation in the area. Considering the underlying social and economic nature of schistosomiasis such as focusing on decreasing the risk of exposure among high-risk occupations is needed to improve the strategies for control and elimination of schistosomiasis.

Similar to schistosomiasis, results showed that the STH prevalence failed to reach the global targets set by the WHO [25]. Results also showed significantly higher prevalence in SAC than adults which is similar with previous studies [24, 26–30]. The risky behavior of children such as playing in the dirt, walking barefoot, and poor hygiene practices are among the reasons why STH prevalence is consistently higher among them than adults. Furthermore, the results of the regression analysis showed that good practices reduce the risk of infection for STH. This provides evidence on the need for health education focusing on promotion of good practices (i.e., sanitation and hygiene) and behavior change to supplement existing control programs for STH.

Results also showed that there is significant variability in the prevalence of intestinal helminthiasis across all locations. Previous studies showed the differences in the environment significantly influence disease transmission [22–24]. The differences in topography, land use, rainfall, and climate were observed to influence transmission of these parasites. For example, low-land areas, flood-prone areas, and agricultural areas are favorable for disease transmission. Further, areas that are near water bodies or wetlands are also favorable for disease transmission especially when they serve as sources of livelihood or entertainment [22–24, 31]. Strengthened surveillance system in villages and spatiotemporal mapping at a granular level is recommended to identify and address environmental factors favoring transmission of these parasites [7]. Moreover, prevalence was consistently higher in the province of Surigao del Norte, specifically in the municipality of San Isidro. Looking at the conditions of the areas in this province, results of the survey showed significantly higher number of unemployed and farmers in Surigao del Norte than in Agusan del Sur. There is also a significantly higher number of households that are exposed to domestic and farm animals in this province. Furthermore, there is also significantly lower sanitary toilet coverage in Surigao del Norte. These factors could have contributed to the observed high prevalence in the province. These differences in the environmental and economic conditions of the areas suggest the need for a more tailor-fitted approach in the implementation of control strategies. At present, implementation of the control strategies is the same throughout the region. A customized approach depending on the needs of the areas would provide higher impact of the control strategies as well as allow cost-effective use of limited resources in the country.

Results of the study showed that intestinal helminthiases remain as public health problem in the country despite ongoing control programs for more than a decade. The control of intestinal helminthiases in both provinces is focused on MDA and improvements in WASH facilities. Records show that the MDA coverage rates in these areas (90–100%) meet the national and global targets, however, observed prevalence in these areas may indicate the need to consider having rapid coverage assessments to validate reported MDA coverage rates [32]. During our conversations with program heads, they mentioned that challenges in MDA include the preference of many for case finding and treatment (i.e., diagnosis first prior to treatment) over mass treatment (i.e., treatment without diagnosis). This is further intensified by fear of adverse reactions to drugs which was exacerbated by the previous vaccine scare in the Philippines affecting numerous public health programs in the country [33]. Nevertheless, identifying and addressing the factors related to MDA coverage rates is needed. House-to-house delivery of drugs, improving health awareness through health education, and ensuring sufficient drug supplies may help in improving MDA coverage rates [34] and continuous efforts must be made to further enhance MDA coverage rates. Moreover, the continuing challenges with safe WASH may have contributed to the persistence of these infections in the area. Records show that sanitary toilet coverages in the selected communities (80.4–96.3%) have failed to reach the national target (100%) [10] which agrees with the results of the household survey. The low sanitary toilet coverage as well as the observed continuing open defecation may have contributed to persisting prevalence of schistosomiasis and STH [35]. Improvements in the accessibility of safe WASH facilities are recommended as these have led to reductions in the prevalence of schistosomiasis and STH, leading to the interruption of transmission of these infections [28]. Furthermore, the majority of households are closely living with animals, which are known reservoirs of intestinal helminths [1]. However, the region lacks control strategies directed to animals. Complementary public health strategies, such as veterinary public health, is recommended to address the zoonotic transmission of these parasites [36].

The persistently high intestinal helminthiases prevalence in these areas despite ongoing control programs could also mean that some barriers to the implemented interventions exist. This study also assessed the knowledge, attitudes, and practices of the residents to obtain a view on their perspectives regarding these intestinal helminthiases. Results showed high awareness of the existence of intestinal helminthiases but generally low knowledge of its transmission and signs and symptoms. Many were unaware of the zoonotic transmission of these parasites and other signs and symptoms, such as pruritus ani, rashes, and loss of appetite, suggestive of these infections. These lack of knowledge and awareness could hamper effective control strategies. As seen in the results of this study, households who have higher awareness are more inclined to participate in local control programs. Thus, strengthening health education in the area through more intensive campaigns (i.e., house-to-house visits, seminars, and community talks) may be needed to raise their awareness on these intestinal helminthiases and raise their compliance to control programs and to consequently promote behavioral change [22, 37–39].

Good prevention practices were observed to be high in the area. However, results also showed a significant negative association between knowledge and practices. Socio-economic factors may have contributed to this finding. For example, evidence showed that farmers are generally aware that tending the rice fields may increase the odds of contracting intestinal helminthiasis, however, they continue to do so and risk being infected rather than losing income [40]. This further highlights the need to understand the underlying social and economic nature of diseases. Considering these factors in the formulation of control strategies may lead to a more sustainable implementation of control programs.

## Conclusions

Results of the study showed remaining high endemicity of intestinal helminthiases in the study area despite ongoing control programs. Poor socio-economic conditions and low awareness about how intestinal helminthiases are transmitted may be among the factors hindering success of intestinal helminth control programs in the provinces of Agusan del Sur and Surigao del Norte. Overall, controlling and eliminating intestinal helminthiases as public health problems also require addressing poverty-related issues. Addressing these sustainability gaps could contribute to the success of alleviating the burden of intestinal helminthiases in endemic areas.

## Electronic supplementary material

Below is the link to the electronic supplementary material.

Supplementary Material 1

## Data Availability

Data supporting this study are not publicly available for ethical reasons. Please contact vvpaller@up.edu.ph or m.betson@surrey.ac.uk.
