# Supplementary material for: Socio-economic risk factors for intestinal helminthiases in selected endemic communities in Mindanao, the Philippines: a cross-sectional study
Source: BMC Infect Dis. 2024 Sep 19;24:1012. doi: 10.1186/s12879-024-09780-5 (PMC11414249; doi:10.1186/s12879-024-09780-5)
Supplement: Supplementary file 1 — Supplementary Material 1 [file 12879_2024_9780_MOESM1_ESM.docx]

**Supplementary Table.** Risk factors associated with intestinal helminth infection in Agusan del Sur and Surigao del Norte, the Philippines

| **Outcomes/Variables** | **Odds Ratio** | **95% CI** | ***p* value** |
| --- | --- | --- | --- |
| **Risk factors associated with schistosomiasis** |  |  |  |
| Municipality (baseline Bunawan) |  |  |  |
| *Trento* | 0.41 | 0.11-1.55 | 0.190 |
| *Mainit* | 0.35 | 0.10-1.29 | 0.114 |
| *San Isidro* | 3.49 | 1.39-8.78 | 0.008^*^ |
| *Bayugan City* | *dropped as it predicts failure perfectly* | | |
| *Esperanza* | 0.66 | 0.10-4.25 | 0.661 |
| *Claver* | 0.15 | 0.01-1.59 | 0.116 |
| *Gigaquit* | 0.61 | 0.13-2.96 | 0.540 |
| Educational attainment (baseline tertiary level) |  |  |  |
| *Primary level* | 1.40 | 0.37-5.32 | 0.623 |
| *Secondary level* | 0.71 | 0.18-2.74 | 0.615 |
| *Vocational training* | *dropped as it predicts failure perfectly* | | |
| *No schooling* | *dropped as it predicts failure perfectly* | | |
| Father’s occupation (baseline unemployed) |  |  |  |
| *Farming* | 1.25 | 0.44-3.50 | 0.673 |
| *Others* | 1.17 | 0.35-3.87 | 0.797 |
| Access to toilet | 1.64 | 0.45-5.91 | 0.449 |
| Kind of toilet (baseline no toilet) |  |  |  |
| *Flush toilet* | *dropped as it predicts failure perfectly* | | |
| *Poor flush toilet* | 0.24 | 0.01-4.44 | 0.337 |
| *Pit latrine* | 0.68 | 0.04-12.65 | 0.798 |
| *Hanging latrine* | *dropped as it predicts failure perfectly* | | |
| Number of cattle in a household *(continuous)* | 1.60 | 0.50-5.11 | 0.425 |
| Previous diagnosis of any intestinal helminthiases | 0.69 | 0.31-1.52 | 0.354 |
| Exposure to rice fields | 2.82 | 0.99-7.99 | 0.051 |
| Bathes in rivers and other bodies of water | 1.29 | 0.47-3.51 | 0.616 |
| ‘Knowledge’ of schistosomiasis score *(continuous)* | 1.02 | 0.80-1.30 | 0.874 |
| ‘Practice’ score *(continuous)* | 1.10 | 0.74-1.64 | 0.649 |
| **Risk factors associated with STH** |  |  |  |
| Municipality (baseline Bunawan) |  |  |  |
| *Trento* | 0.96 | 0.31-2.92 | 0.941 |
| *Mainit* | 3.89 | 1.65-9.19 | 0.002^*^ |
| *San Isidro* | 11.98 | 4.62-31.02 | <0.001^*^ |
| *Bayugan City* | 2.93 | 1.09-7.87 | 0.033^*^ |
| *Esperanza* | 2.49 | 1.01-6.19 | 0.049^*^ |
| *Claver* | 1.08 | 0.27-4.37 | 0.916 |
| *Gigaquit* | 0.88 | 0.25-3.08 | 0.848 |
| Educational attainment (baseline tertiary level) |  |  |  |
| *Primary level* | 3.76 | 1.45-9.72 | 0.006^*^ |
| *Secondary level* | 2.80 | 1.12-7.02 | 0.028^*^ |
| *No schooling* | *dropped as it predicts failure perfectly* | | |
| Father’s occupation (baseline unemployed) |  |  |  |
| *Farming* | 1.92 | 0.93-3.96 | 0.077 |
| *Others* | 1.40 | 0.64-3.04 | 0.395 |
| Presence of rodents | 2.24 | 0.89-5.64 | 0.085 |
| Previous diagnosis of any intestinal helminthiases | 1.21 | 0.64-2.28 | 0.551 |
| Exposure to rice fields | 0.61 | 0.31-1.20 | 0.154 |
| Bathes in rivers and other bodies of water | 1.07 | 0.54-2.11 | 0.855 |
| Walking barefoot | 1.06 | 0.55-2.04 | 0.863 |
| ‘Knowledge’ of STH score *(continuous)* | 0.87 | 0.71-1.07 | 0.187 |
| ‘Practice’ score *(continuous)* | 0.84 | 0.70-0.99 | 0.044^*^ |

*^*^significant at 5% level of significance*
